# Exploring first-degree family history in a cohort of Portuguese Alzheimer’s disease patients: population evidence for X-chromosome linked and recessive inheritance of risk factors

**DOI:** 10.1007/s00415-024-12673-x

**Published:** 2024-09-05

**Authors:** Miguel Tábuas-Pereira, Catarina Bernardes, João Durães, Marisa Lima, Ana Rita Nogueira, Jorge Saraiva, Teresa Tábuas, Mariana Coelho, Kimberly Paquette, Kaitlyn Westra, Célia Kun-Rodrigues, Maria Rosário Almeida, Inês Baldeiras, José Brás, Rita Guerreiro, Isabel Santana

**Affiliations:** 1 Faculty of Medicine, University of Coimbra, Coimbra, Portugal; 2 Centro Hospitalar e Universitário de Coimbra, Coimbra, Portugal; 3 Centre for Innovative Biomedicine and Biotechnology (CIBB), Universidade de Coimbra, Coimbra, Portugal; 4 Instituto Politécnico de Bragança, Bragança, Portugal; 5 Department of Neurodegenerative Science, Van Andel Institute, Grand Rapids, MI USA; 6 Neurology Department, Centro Hospitalar e Universitário de Coimbra, Praceta Prof. Mota Pinto, 3004-561 Coimbra, Portugal

**Keywords:** Family, Alzheimer, X-chromosome, Homozygosity, Consanguinity

## Abstract

**Background:**

Alzheimer’s disease (AD) heritability is estimated to be around 70–80%. Yet, much of it remains to be explained. Studying transmission patterns may help in understanding other factors contributing to the development of AD.

**Objective:**

In this study, we aimed to search for evidence of autosomal recessive or X- and Y-linked inheritance of risk factors in a large cohort of Portuguese AD patients.

**Methods:**

We collected family history from patients with AD and cognitively healthy controls over 75 years of age. We compared the proportions of maternal and paternal history in male and female patients and controls (to search for evidence of X-linked and Y-linked inherited risk factors). We compared the risk of developing AD depending on parents’ birthplace (same vs. different), as a proxy of remote consanguinity. We performed linear regressions to study the association of these variables with different endophenotypes.

**Results:**

We included 3090 participants, 2183 cognitively healthy controls and 907 patients with AD. Men whose mother had dementia have increased odds of developing AD comparing to women whose mother had dementia. In female patients with a CSF biomarker-supported diagnosis of AD, paternal history of dementia is associated with increased CSF phosphorylated Tau levels. People whose parents are from the same town have higher risk of dementia. In multivariate analysis, this proxy is associated with a lower age of onset and higher CSF phosphorylated tau.

**Conclusions:**

Our study gives evidence supporting an increased risk of developing AD associated with an X-linked inheritance pattern and remote consanguinity.

**Supplementary Information:**

The online version contains supplementary material available at 10.1007/s00415-024-12673-x.

## Introduction

Alzheimer’s disease (AD) stands as the most prevalent neurodegenerative disorder. Its heritability is estimated at 70–80% [1, 2]. Despite this, only three genes (*APP, PSEN1*, and *PSEN2*) are associated with known pathogenic variants transmitted in a Mendelian autosomal dominant manner, accounting for less than 1% of cases [3]. The *APOE* gene remains the primary identified genetic risk factor, contributing to an estimated 9.3% of the heritability [1]. Additionally, numerous genes with common variants of small effect have been unveiled through increasingly expanding genome-wide association studies (GWAS). Nonetheless, a considerable portion of AD heritability remains unexplained [1, 4].

Hence, it is hypothesized that rare variants with moderate to large impact may explain much of this missing heritability and be the major factor driving the development in AD in many families [5]. However, some other factors may explain the missing heritability, such as effects like epistasis [6] and gene-environment interaction [7], the fact that GWAS have caveats in ascertaining heritability [8], or the exclusion of sexual chromosomes in past GWAS [9]. Notably, the investigation of X- and Y-linked heritability remains underexplored, alongside the potential contribution of homozygous risk factors to the inherited risk of AD [10].

In fact, a positive family history is a recognized risk factor for AD [11], with genetics being the principal contributor to that risk [12]. Studying family history and investigating transmission patterns across diverse populations may shed light on additional factors influencing AD development. In this study, we aim to investigate evidence for autosomal recessive or X- and Y-linked inheritance patterns of risk genetic variants within a large cohort of Portuguese Alzheimer’s disease patients.

## Methods

### Participant selection

We collected patients from a convenience sample of patients from a cohort of AD patients followed at the Dementia Clinic, Neurology Department of University Hospital of Coimbra, Portugal. This center serves as the tertiary referral hospital in the central region of Portugal, comprising around 2.2 million people. All patients underwent a thorough diagnostic investigation including standard clinical evaluation, neuropsychological assessment, laboratory analysis, and imaging studies. Per routine, patients under 75 years of age at the first evaluation are proposed to CSF collection or amyloid-PET. AD diagnosis was established by the most recent criteria [13, 14]. Patients were considered to have early-onset AD (EOAD) if the age of onset was of 65 or lower, and late-onset AD (LOAD), if the age of onset was 66 or higher.

Controls were collected in the hospital (patients for other non-neurological conditions and relatives that came with patients to the clinic) and nursing homes, all with a normal MMSE (according to the Portuguese normative data [15]) and with collateral history of normal cognitive status. To decrease the odds of future dementia, only subjects with more than 75 years of age at the time of the collection were included. Subjects with a diagnosis (or suspicion of) of Parkinson’s disease or other form of degenerative parkinsonism, Frontotemporal dementia or other form of dementia, amyotrophic lateral sclerosis or other form of neurodegenerative disorder were excluded.

Family history was collected with subjects and relatives, through a systematic questionnaire. The questionnaire asked for place of birth of the father and the mother, age of death, cause of death (if known), history of dementia, and age of onset of the dementia, number of siblings, number of sisters and brothers, age of death (if dead), cause of death, history of dementia in any of the siblings, age of onset of the dementia.

### APOE and CSF analysis

For *APOE* genotyping, DNA was isolated from whole EDTA-blood using a commercial kit (Roche Diagnostics GmbH, Manheim, Germany) and *APOE* genotype was determined by polymerase chain reaction-restriction fragment length polymorphisms assay [16]. CSF was obtained by lumbar puncture and biomarkers (Aβ42, t-tau and p-tau) measurements were performed as previously reported by Baldeiras et al. [17]. Pre-analytical and analytical procedures were done in accordance with the Alzheimer’s Association guidelines for CSF biomarkers determination [18]. External quality control of the assays was performed under the scope of the Alzheimer’s association quality control program for CSF biomarkers [18]. CSF biomarkers were classified as normal/abnormal according to previously reported laboratory reference values [19].

### Analysis

#### Y-chromosome linked inheritance

To study a possible link of the genetic risk of AD and variants in the Y chromosome, we compared (1) the proportion of AD versus controls in male and female subjects with paternal history of dementia. The hypothesis is that if part of the risk is linked with the Y chromosome, male descendants would have a higher proportion of dementia. We also compared (2) the proportion of AD in males with paternal history of dementia with all females with positive parental history of dementia (to increase power) and (3) males with maternal history of dementia (to account for differential risk of AD depending on sex). Comparisons with other dyads are also shown to better frame these results.

#### X-chromosome linked inheritance

To investigate a possible link of the genetic risk of AD and variants in the X chromosome, we compared (1) the proportion of patients with AD in females whose mothers had dementia and in males whose mother had dementia. The hypothesis is that due to random X-chromosome inactivation, X linked risk will be attenuated in women. We also compared (2) male patients with maternal history of dementia with female patients with paternal history of dementia; (3) male patients with all females with parental history of dementia. Comparisons with other dyads are also shown to better frame these results.

In female patients with AD, we also studied the association of maternal and paternal history of dementia with some AD endophenotypes (age of onset, CSF Amyloid-Beta_42_, CSF total-Tau and CSF phosphorylated-Tau), adjusted for other variables.

### Remote consanguinity

To investigate the possible role of homozygous factors underlying the risk of AD, we studied the risk in participants whose parents were born in the same place with those whose parents were born in different places. Places that were considered different towns or villages, although neighboring each other, were considered as different. We compared both groups in terms of risk of AD and the relationship with some AD endophenotypes (age of onset, CSF Amyloid-Beta_42_, CSF total-Tau and CSF phosphorylated-Tau).

### Statistical analysis

Statistical analysis was performed using IBM SPSS Statistics software version 26. Normality was ascertained using the Kolmogorov–Smirnov test. Categorical variables are represented using frequencies and were compared through Chi-square tests. Ordinal or discrete variable are reported as means as they are better perceived, but were studied using median values and were compared through Mann–Whitney *U* tests. Subjects with both parents with dementia were disregarded when univariate analysis was performed, but included when performing linear regressions.

To compare the different parent-descendant dyads (as described in the previous paragraphs) to assess differential risk of developing AD, we compared the proportions of patients and controls who had positive or negative paternal/maternal history of dementia. From those we calculated odds and then odds ratios. From odds ratios, we calculated significance, according to the following equation:

$$z=\frac{\text{ln}\left(\Phi \right)-0}{\sqrt{\frac{1}{np(1-p)}+\frac{1}{{n}{\prime}p{\prime}(1-p{\prime})}}},$$

where *Φ* is the odds ratio, n and n’ are the samples sizes and *p* and *p*′ are the proportions.

We performed different linear regressions in the subset of female AD patients with CSF biomarkers to study relationships between paternal and maternal history and these endophenotypes. We chose to study only female, because it was the larger group, giving higher power to detect associations. We performed a logistic regression to determine if having parents with the same birthplace is associated with AD risk. To probe for support of this relationship from biological markers, we studied this in the linear regression models abovementioned, which included only female AD patients with CSF biomarkers.

All the assumptions of these models were verified. Alpha was set for 0.05. Due to the exploratory nature of our study, we did not correct for multiple comparisons.

### Ethics

The present research complied with the ethical guidelines for human experimentation stated in the Declaration of Helsinki and was approved by the Ethics Board of Coimbra University Hospital. An informed consent was obtained from all the participants after the aims and procedures of investigation were fully explained by a member of the study group.

## Results

We included 3090 participants, 2183 cognitively healthy controls and 907 patients with AD. Of the patients, 237 (25.5%) were diagnosed with EOAD and 670 (74.3%) with LOAD. *APOE* polymorphism status was determined for 1667 (76.4%) of the controls and 836 (92.2%) of the AD patients. It was possible to ascertain paternal history of cognitive status from 3006 subjects and maternal history of cognitive status from 3055 subjects. In others, either the father was unknown, subjects lost connection with the parents, or family history could not be recollected by the relatives (and patients could not recollect this information accurately). For 2745 (1838 controls and 907 patients) local of birth of both parents was collected.

In the patients, 331 had CSF biomarkers data and 102 had amyloid PET status. Other demographic variables, *APOE* and family history are reported in Table 1.

**Table 1:** Demographic, *APOE* and family history variables of patients and controls

	Controls (*n* = 2183)	AD patients (*n* = 907)	*p*
Female sex (*n*, %)	1408 (64.5)	565 (62.3)	0.250
Education (years ± SD)	3.9 ± 3.5	5.5 ± 4.4	< 0.001
Age of onset (years ± SD)		71.7 ± 9.1	
APOE ∊ 2 ∊ 2 (*n*, %)	6 (0.4)	2 (0.2)	< 0.001
APOE ∊ 2 ∊ 3 (*n*, %)	185 (11.1)	37 (4.4)	
APOE ∊ 2 ∊ 4 (*n*, %)	15 (0.9)	13 (1.6)	
APOE ∊ 3 ∊ 3 (*n*, %)	1210 (72.6)	394 (47.1)	
APOE ∊ 3 ∊ 4 (*n*, %)	240 (14.4)	311(37.2)	
APOE ∊ 4 ∊ 4 (*n*, %)	11 (0.7)	79 (9.5)	
Positive family history of dementia (*n*, %)	414 (19.4)	417 (48.8)	< 0.001
Positive paternal family history of dementia (*n*, %)	100 (4.7)	116 (13.5)	< 0.001
Positive maternal family history of dementia (*n*, %)	193 (8.9)	232 (26.3)	< 0.001
Siblings with dementia (*n*, %)	178 (8.2)	208 (23.0)	< 0.001
Parents from the same town (*n*, %)	1275 (58.4)	543 (60.0)	0.470
CSF amyloid-Beta_42_ (pg/mL ± SD)		516.8 ± 189.1	
CSF total-Tau (pg/mL ± SD)		637.9 ± 346.2	
CSF phosphorylated-Tau (pg/mL ± SD)		105.9 ± 62.7	

*AD* Alzheimer’s disease, *SD* standard deviation, *APOE* apolipoprotein E, *CSF* cerebrospinal fluid

Positive family history is far more frequent in patients (EOAD and LOAD). Both EOAD and LOAD patients have a positive family history in around half of the cases. Positive maternal history of dementia is more frequent in EOAD than in LOAD, whereas positive paternal history is similar. LOAD patients have more frequently a sibling with dementia (supplemental Table 1).

Having positive family history (both maternal and paternal) seems to lead to a larger increase in risk of developing AD in men than in women. However, we did not find statistically significant differences in terms of AD risk in Y-chromosome related dyads (father–son) versus the other dyads. Male subjects with maternal history of dementia have a larger increase in the risk of developing AD than that of their female counterparts (Table 2). To better capture this relationship, we performed linear regressions for different endophenotypes in female AD patients (Table 3). Paternal history (but not maternal) is associated with reduced CSF tau and increased phosphorylated-tau (Table 3).

**Table 2:** Comparison of AD risk in Y-chromosome inheritance relationships (father–son) with different X-chromosome inheritance dyads and comparison of the AD risk between mother–son and other X-chromosome related dyads (Φ is the relative odds ratio, in bold where p<0.05)

	Controls	Alzheimer’s disease	Relative risk	Comparisons						
				Father → son	Father → daughter	Any parent → daughter	Mother → daughter	Mother → son	Any parent → son	Any parent → any child
Father → son	36/761 (0.047)	52/325 (0.16)	3.38							
Father → daughter	64/1383 (0.046)	64/537(0.119)	2.58	*Φ* = 0.76, *p* = 0.095						
Any parent → daughter	189/1382 (0.137)	184/533 (0.345)	2.52	*Φ* = 0.746 * p* = 0.073	*Φ* = 0.980. * p* = 0.822					
Mother → daughter	134/1404 (0.095)	140/551 (0.254)	2.66	*Φ* = 0.787, * p* = 0.142	*Φ* = 1.034, * p* = 0.807	*Φ* = 1.054, * p* = 0.695				
Mother → son	59/768 (0.077)	92/332 (0.277)	3.72	*Φ* = 1.100, * p* = 0.617	***Φ* = 1.444, *p* = 0.028**	***Φ* = 1.473, *p* = 0.020**	***Φ* = 1.397, *p* = 0.045**			
Any parent → son	90/757 (0.113)	128/322 (0.398)	3.34	*Φ* = 0.989, * p* = 0.951	*Φ* = 1.298, * p* = 0.111	*Φ* = 1.325, * p* = 0.086	*Φ* = 1.256, * p* = 0.163	*Φ* = 0.899, * p* = 0.575		
Any parent → any child	279/2139 (0.130)	312/855 (0.365)	2.80	*Φ* = 0.827, * p* = 0.206	*Φ* = 1.086, * p* = 0.503	*Φ* = 1.108, * p* = 0.405	*Φ* = 1.051, * p* = 0.687	*Φ* = 0.752, * p* = 0.070	*Φ* = 0.837, * p* = 0.245

**Table 3:** Linear regression models to test the association of maternal and paternal history of dementia in women and different outcome variables (age of onset, CSF amyloid-Beta_42_, CST TOTAL-Tau and CSF phosphorylated Tau), adjusting for different covariates

	Age of onset (*β*, 95% CI, *p*)^a^	CSF amyloid-Beta_42_ (*β*, 95% CI, *p*)^b^	CST total-Tau (*β*, 95% CI, *p*)^c^	CSF phosphorylated Tau (β, 95% CI, *p*)^d^
Paternal history of dementia	− 0.175, [− 1.785, 1.435], *p* = 0.830	51.674, [− 31.522, 134.871], * p* = 0.222	**− 99.295, [− 168.961, − 29.628], *p* = 0.006**	**19.302, [6.305, 32.300], *p* = 0.004**
Maternal history of dementia	0.561, [− 0.626, 1.747], * p* = 0.352	49.727, [− 11.542, 110.996], * p* = 0.111	− 14.178, [− 66.967, 38.611], * p* = 0.596	− 0.750, [− 10.628, 9.128], * p* = 0.881
Parents from the same town	**− 1.447, [− 2.500, − 0.393], *p* = 0.007**	**71.505, [16.674, 126.335], *p* = 0.011**	**− 69.366, [− 115.948, − 22.784], *p* = 0.004**	**4.135, [21.563,], *p* = 0.004**
Age at LP	0.000, [− 0.001, 0.000], * p* = 0.350	0.011, [− 0.008, 0.029], * p* = 0.258	− 0.006, [− 0.022, 0.010], * p* = 0.430	0.001, [− 0.002, 0.004], * p* = 0.403
Education	0.026, [− 0.069, 0.122], * p* = 0.587	0.125, [− 4.832, 5.081], * p* = 0.960	0.316, [− 3.921, 4.554], * p* = 0.883	− 0.163, [− 0.954, 0.629], * p* = 0.686
Age of onset	–	**16.243, [8.260, 24.226], *p* < 0.001**	**− 11.453, [− 18.397, − 4.510], *p* = 0.001**	**2.337, [1.048, 3.627], *p* < 0.001**
APOE	− 1.572, [− 2.665, − 0.479], * p* = 0.005	− 33.486, [− 91.504, 24.532], * p* = 0.256	− 37.637, [− 87.088, 11.815], * p* = 0.135	**9.266, [0.072, 18.460], *p* = 0.048**
CSF amyloid-Beta_42_	**0.006, [0.003, 0.009], *p* < 0.001**	–	**0.153, [0.016, 0.289], *p* = 0.028**	− 0.006, [− 0.032, − 0.020], * p* = 0.635
CST total-Tau	**− 0.006, [− 0.009, − 0.002], *p* = 0.001**	**0.209, [0.022, 0.395], *p* = 0.028**	–	**0.171, [0.159, 0.183], *p* < 0.001**
CSF phosphorylated Tau	**0.034, [0.015, − 0.053], *p* < 0.001**	− 0.244, [− 1.256, 0.768], * p* = 0.635	**4.899, [4.552, 5.247], *p* < 0.001**	–
Year of birth	**− 0.888, [− 0.956, − 0.820], *p* < 0.001**	**13.305, [5.329, 21.280], *p* = 0.001**	**− 8.615, [− 15.539, − 1.690], *p* = 0.015**	**1.991, [0.710, 3.272], *p* = 0.003**

*AD* Alzheimer’s disease, *APOE* apolipoprotein E, *CSF* cerebrospinal fluid

^a^F(10, 149) = 78.826, *p* < 0.001, *R*^2^ = 0.841

^b^(10, 149) = 6.106, *p* < 0.001, *R*^2^ = 0.291

^c^(10, 149) = 95.853, *p* < 0.001, *R*^2^ = 0.865

^d^(10, 149) = 98.263, *p* < 0.001, *R*^2^ = 0.868

(Φ is the relative odds ratio, in bold where p<0.05)

Concerning the remote consanguinity hypothesis, having parents from the same town increases the odds for AD (Tables 3, 4). This association was further investigated in the linear regression models abovementioned (Table 3) and they revealed an association of this proxy with lower age of onset and higher CSF-phosphorylated tau, but higher CSF Amyloid-Beta_42_ and lower tau.

**Table 4:** Logistic regression for demographic variables and risk of developing Alzheimer’s disease (*γ*^2^(5, 2663) = 1448.749, -2 log likelihood = 1955.493, Nagelkerke *R*^2^ = 0.581)

Variable	OR	95% confidence interval	*p*
Sex	1.040	0.826, 1.310	0.738
Age of onset	0.768	0.750, 0.785	< 0.001
Education	0.978	0.948, 1.009	0.169
APOE (≥ 1 ε4 allele)	1.556	1.255, 1.930	< 0.001
Parents from the same town	1.308	1.042, 1.643	0.021

*OR* odds ratio, *APOE* apolipoprotein E

## Discussion

We report on a reasonably sized population study, with well-characterized AD patients and controls with an age at collection over 75. Positive parental history of dementia is more common in early-onset cases, especially maternal history. Men with a maternal history of dementia develop AD more frequently than women whose mother had dementia. In female patients with a CSF-supported diagnosis of AD, paternal history of dementia is associated with lower CSF total Tau, but increased CSF phosphorylated Tau. People whose parents are from the same town have higher risk of dementia. In multivariate analysis in AD patients with CSF biomarkers, this proxy is associated with a lower age of onset and higher CSF phosphorylated tau, but lower tau and higher CSF amyloid-Beta_42_.

Concerning the positive family history in EOAD versus LOAD, we found that around half (50.9%) of the EOAD patients have a first degree relative with dementia (with a slightly lower proportion—48.1%—in LOAD patients). EOAD have parental family history more frequently than LOAD. This is driven mostly by maternal history, since EOAD patients have maternal family history more frequently than LOAD patients but paternal family history was comparable between EOAD and LOAD. Parental family history is more common in EOAD than in LOAD, supporting that variants with a larger impact are probably driving most of the risk in EOAD patients. LOAD patients have more siblings with dementia, possibly due to the fact that LOAD patients have older siblings, and this risk may be driven more by age than genetic risk. Men with positive parental history of dementia have a larger increase in odds of developing AD than women with the same history, suggesting that genetics may be more determinant in men than in women.

Women have an increased risk of developing AD [20], which has been attributed to different reasons, such as longer longevity [21] and hormonal differences [21]. A possible role of common risk variants in the X-chromosome could be one explanation for differential sex prevalence [22, 23]. Sexual chromosomes are typically disregarded in GWAS [9]. Twin studies, although showing higher within-pair correlations for female co-twins, failed to demonstrate sex differences in heritability [24]. Yet, X chromosome inactivation complicates analysis in that setting, masking some of the genetic input. In our study, sons of women with dementia have higher odds of developing AD than daughters of women with dementia. This suggests that some variants in the X-chromosome may carry some AD risk, which may either be recessive or not expressed due to X-inactivation in women, but become apparent in men. Further studies on this matter are of the utmost importance, as they may help to understand why women have an increased risk of AD, and may lead to new therapeutic targets, especially important to this higher risk group. Interestingly, in women with AD, paternal family history (but not maternal) was associated with higher CSF phosphorylated-tau levels, but reduced total tau-levels. This suggests a link between the X-chromosome variants and tau phosphorylation pathways, with patients with paternal history having less tau, but with increased levels of tau phosphorylation. These findings are in line with some other related findings, such as the X-chromosome being associated with tau pathology [25] and the fact that there is an apparent sex-specific modulation of tau phosphorylation [26, 27].

We did not find an association between Y-chromosome inheritance and the risk of AD. However, male patients with paternal family history of dementia developed AD more frequently than women with the paternal family history. This relationship may be masked by the higher prevalence of dementia in women, so it is possible (even likely) there is an association, but our study was underpowered.

In this study, we used the birthplace of the parents as a proxy of autozygosity. Having the same birthplace suggests that the parents’ genetic pool would be limited, which will lead to homozygosity enrichment [28]. APOE results were in line with this, as people with parents with the same birthplace had ∊ 2 ∊ 2 and ∊ 4 ∊ 4 more frequently (supplemental Table 2). Previous studies with genetic data on large populations demonstrated that autozygosity and runs of homozygosity [10] are associated with the risk of dementia [29]. We further demonstrate that this proxy is associated not only with a higher risk of dementia, but also with a lower age of onset and higher levels of phosphorylated tau, endophenotypes of the disease. Yet, we found an inverse relationship with tau and CSF Aβ_42_. This could mean that the main homozygous factors in our population are more associated with Tau-related than amyloid-related pathways, or that patients with autozygosity may have increased burden in other steps of the pathophysiology of the disease, developing disease before the levels of CFB Aβ_42_ decrease as much.

Our study has some limitations. First, we draw hypothesis from population data and not genetic data. Family history was collected through the family and may be biased. We could not ascertain for true paternity. Remote consanguinity was evaluated through parents’ place of birth, a rough measure, especially prone to error when both parents are from the same city. The associations were not controlled for possible confounders, such as parental socioeconomic status, parental education or others. Many of our associations were also supported with further associations with endophenotypes in the CSF biomarker supported patients, giving it additional strength. However, we collected a large cohort of subjects, carefully evaluated by an experienced team, with biomarker-supported diagnosis in a large proportion of the patients.

In conclusion, in a reasonably large study of the Portuguese population, we give indirect evidence supporting the existence of genetic risk variants in the X-chromosome and autosomal recessive alleles carrying AD risk. This stresses the importance of adequate study of genetic variants in the sex chromosomes in Alzheimer’s disease.

## Supplementary Information

Below is the link to the electronic supplementary material.

Supplementary file1 (DOCX 18 KB)

Supplementary file2 (DOCX 22 KB)

## Data Availability

Data is available by request to the authors or in the NIH repository.
