# Supplementary material for: Exploring first-degree family history in a cohort of Portuguese Alzheimer’s disease patients: population evidence for X-chromosome linked and recessive inheritance of risk factors
Source: J Neurol. 2024 Sep 5;271(10):6983–90. doi: 10.1007/s00415-024-12673-x (PMC11447147; doi:10.1007/s00415-024-12673-x)
Supplement: Supplementary file 1 — Supplementary file1 (DOCX 18 KB) [file 415_2024_12673_MOESM1_ESM.docx]

**Supplemental Tables**

**Supplemental Table 1**: Comparison of positive maternal, paternal and fraternal family history in Early-Onset vs Late-Onset Alzheimer’s Disease patients.

| **Family History** | **Controls** | **AD patients** | **p** |
| --- | --- | --- | --- |
| **Paternal Family History in EOAD (n, %)** | 100 (4.7) | 29 (12.8) | **<0.001** |
| **Paternal Family History in LOAD (n, %)** | 100 (4.7) | 87 (13.7) | **<0.001** |
| **LOAD vs EOAD** | | | 0.726 |
| **Maternal Family History in EOAD (n, %)** | 193 (8.9) | 81 (34.9) | **<0.001** |
| **Maternal Family History in LOAD (n, %)** | 193 (8.9) | 151 (23.2) | **<0.001** |
| **LOAD vs EOAD** | | | **<0.001** |
| **Fraternal Family History in EOAD (n, %)** | 178 (8.2) | 36 (15.3) | **<0.001** |
| **Fraternal Family History in LOAD (n, %)** | 178 (8.2) | 172 (25.6) | **<0.001** |
| **LOAD vs EOAD** | | | **0.001** |
| **First-degree Family History in EOAD (n, %)** | 0 (0.0) | 114 (50.9) | **<0.001** |
| **First-degree Family History in LOAD (n, %)** | 414 (19.4) | 303 (48.1) | **<0.001** |
| **LOAD vs EOAD** | | | 0.472 |

EOAD: Early-Onset Alzheimer’s Disease; LOAD: Late-Onset Alzheimer’s Disease.

**Supplemental Table 2**: APOE genotypes in subjects whose parents were born in the same place and whose parents whore born in different towns (Χ^2^= 7.645, p=0.265).

| **Apolipoprotein E genotype** | **From the same town** | **Not from the same town** | **Total** |
| --- | --- | --- | --- |
| **∊2∊2** | 2 | 0 | 2 |
| **∊2∊3** | 22 | 15 | 37 |
| **∊2∊4** | 11 | 2 | 13 |
| **∊3∊3** | 224 | 170 | 394 |
| **∊3∊4** | 191 | 120 | 311 |
| **∊4∊4** | 47 | 32 | 79 |
| **Total** | 543 | 363 | 907 |
